# A Study of Comparison of Outcomes of Submucous Diathermy, Coblation and Micro-debrider Assisted Inferior Turbinoplasty in Patients Having Inferior Turbinate Hypertrophy

**DOI:** 10.1007/s12070-024-04501-5

**Published:** 2024-03-01

**Authors:** Priyanka Ramakrishna Bhagat, Meeta Bathla, Hiren Doshi, Karnadev Solanki, Ritiksha Gajjar

**Affiliations:** 1 Department of Otorhinolaryngology, Narendra Modi Medical College, 59, Bungalow Area, Near Sona Park, Kubernagar, Ahmedabad, 382340 India; 2 I-6 Maruti Nandan Villa -1, Near Govt. Tube 0077Ell, Bhopal, Ahmedabad, 380058 India; 3 Department of Otorhinolaryngology, Narendra Modi Medical College, A/501, Felicia Appartment, Behind Vardan Tower, Naranpura, Ahmedabad, 380013 India; 4 Department of Otorhinolaryngology, Narendra Modi Medical College, A/4 Sitabaug Society, Near Jaihind Char Rasta, Manianagr, Ahmedabad, 380008 India; 5 C/8 Jay Ambe Apartment, Gurudwara Road, Opposie Railway Club, Maninagar, Ahmedabad, 380008 India

**Keywords:** SNOT22 score, Endoscopic grading, Symptomatic improvement, Intra-operative timing and bleeding, Post-operative crusting, Post-operative VAS (Visual analogue scale) pain score

## Abstract

The cause of nasal obstruction in most of the patients is either nasal septal deviation or turbinate hypertrophy owing to vasomotor or perennial allergic rhinitis. Most cases of hypertrophic turbinate are usually mild and respond to antihistamine therapy, local decongestions, or allergy desensitization; however, surgery is required in some cases. In our present study, three surgical methods were used for inferior turbinoplasty i.e. Sub-mucous Diathermy, Coblation and Micro-debrider and patients were divided randomly in these groups. The efficacy and outcomes of these methods was compared on the basis of subjective and objective relief of symptoms and their safety, recurrence and post-operative morbidity. Out of 45 patients, highest number of patients belonged to 20–40 years of age with the mean age of 28.7 years and male female ration 0.78:1. All the patients were evaluated on the basis of preoperative Endoscopic grading of inferior turbinate and SNOT22 symptom scores (Sino Nasal Outcome Test 22), intra-operative timing and bleeding and post-operative pain, crusting, SNOT22 scores (Sino Nasal Outcome Test 22) and Endoscopic grading improvement in inferior turbinate. On comparing all the above methods, we found that Coblation and Micro- debrider were more or less equally effective and better than Sub-mucous diathermy for inferior turbinoplasty. Submucous diathermy has least benefits, still most commonly used method because of its simplicity, conventionality and least cost factor while other two methods need capital investment and higher learning curve of the surgeon.

## Introduction

Nasal obstruction is one of the most common presenting symptoms encountered by otorhinolaryngologists. In most patients, the cause of nasal obstruction is either nasal septal deviation or turbinate hypertrophy owing to vasomotor or perennial allergic rhinitis. Surgical treatment is controversial, and many surgical methods of treatment have been proposed [1]. The enlargement of inferior turbinate can be compensatory hypertrophy, protrusion or hyperplasia of head, tail or whole turbinate [2].

Management usually starts with medical management. Topical nasal steroids are the mainstay of treatment for both allergic and non-allergic rhinosinusitis. Topical steroid is often combined with oral antihistamines, antibiotics and saline in the treatment of chronic rhinosinusitis. Most of the mild and moderate category symptomatic patients get relieved by medical management [2].

When conservative medical management of symptomatically enlarged inferior turbinates is ineffective, the obstructing tissue may be reduced by an intra-mucosal or extra-mucosal destructive procedure (such as electrocautery, cryotherapy, or laser vaporization), or by conservative surgical resection. In my study, we have compared efficacy and outcomes of Submucous diathermy, Coblation and Micro-debrider assisted inferior turbinoplasty.

I have chosen this study because Inferior turbinate hypertrophy is one of the common cause of nasal obstruction which is often associated with other nasal pathologies like Deviated nasal septum (DNS), Chronic Rhinosinusitis (CRS) and Nasal polyposis. So application and evaluation of different types of surgical methods for inferior turbinoplasty can be beneficial and suitable to both the surgeon and the patient as there may not be need of a different surgery.

### Aims

1. To find the method better in terms of objective and subjective improvement in the patients undergoing inferior turbinoplasty by Submucous Diathermy, Coblation and Micro-debrider.
2. To find the most preferred and beneficial method of inferior turbinoplasty.

### Objectives

1. To study the outcomes of Submucous Diathermy, Coblation and Micro-debrider for inferior turbinoplasty.
2. To compare the efficacy of Submucous Diathermy, Coblation and Micro-debrider in terms of both subjective and objective relief of symptoms.
3. To compare the efficacy of Submucous Diathermy, Coblation and Micro-debrider in terms of safety, recurrence and postoperative morbidity.

## Material and Methodology

The present study included all the cases of Inferior turbinate hypertrophy presenting with complaint of nasal blockage, not responding to medical line of management and where surgery was indicated, attending the Otorhinolaryngology department from October 2020 to August 2022.

Medical treatment was given to the patients presenting with the complaint of nasal blockage in the form of nasal and oral decongestants, oral leukotriene inhibitors, intra-nasal corticosteroids and oral corticosteroids for a period of 3–6 months before proceeding for further surgical management for inferior turbinate hypertrophy and other associated nasal pathology.

A total of 45 patients who had clinical features suggestive of Inferior turbinate hypertrophy were evaluated using a standard proforma and underwent the following investigative procedures systematically as and when needed. They were willing to undergo endoscopic turbinoplasty. The patients were randomly distributed for the type of surgical method used for turbinoplasty.

### Inclusion Criteria

1. All the cases of symptomatic inferior turbinate hypertrophy not responding to medical treatment.
2. Individuals of all the age groups.
3. Patients who are willing and fit for surgery.
4. Patients who give consent to participate in the study.

### Exclusion Criteria

1. Patients with severe comorbidities and are unfit for surgery.
2. Patients who are not willing for surgery.

Consent: (i) In the subjects less than 12 years of age their parents gave the informed consent.

(ii) In the subjects from 12 to 18 years of age their parents gave the informed assent.

(iii) In the subjects above 18 years of age their informed consent was taken.

**Fig. 1:**
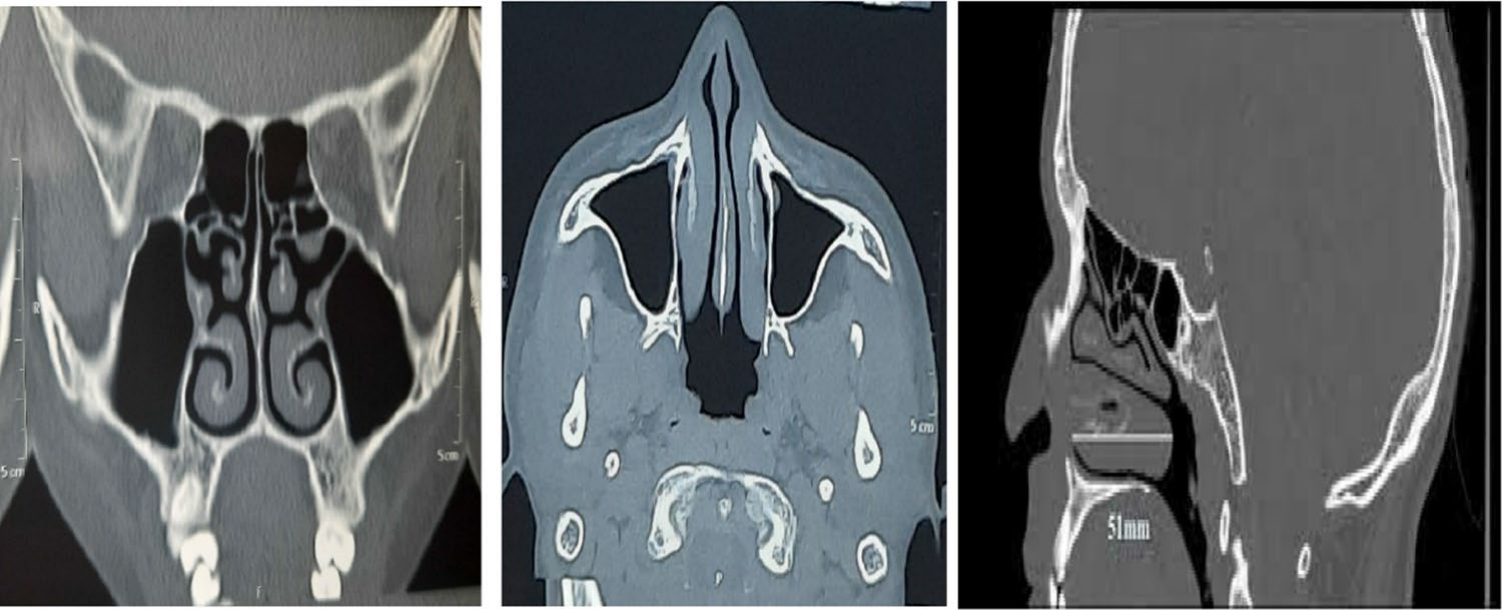
CT PNS (coronal, axial and sagittal sections) showing Bilateral Inferior turbinate hypertrophy (ITH)

*IEC Number:* This study has all approval from the Institutional Review Board (IRB) Ethics committee vide reference number NaMoMC/IRB/2023/62. As per surgical principles all the patients underwent preoperative evaluation in the following aspects: The cases selected for the study were subjected to detailed history taking and examination. A routine haemogram (Hemoglobin, absolute platelet count, Total count, Differential count), blood sugar, urine routine, renal function test, liver function test, X-Ray chest, ECG was done. Each patient underwent local and systemic examination, a systematic diagnostic nasal endoscopy and SNOT 22 (Sino nasal outcome test 22) scoring for their present nasal symptoms. Radiological investigation included a computed tomography of Nose and PNS (paranasal sinuses) (Fig. 1).

### Endoscopic Turbinate Grading

On Nasal endoscopic examination, Status of the Inferior turbinate was done by Camacho et al. grading system [3] as follows:

(A) Grade 1 (0–25% of total airway space).

(B) Grade 2 (26–50% of total airway space).

(C) Grade 3 (51–75% of total airway space).

(D) Grade 4 (76–100% of total airway space).

### Surgical Management

Most patients with inferior turbinate enlargement respond to medical therapy. Surgery is required only for those in whom there is mucosal hypertrophy which is refractory to medical treatment. To achieve complete resolution of nasal symptoms it is mandatory that these coexisting abnormalities (like Deviated nasal septum, Chronic Rhinosinusitis and Allergic rhinitis) be also addressed at the same sitting.

The goal of an ideal turbinate reduction procedure should be optimal volume reduction to diminish symptoms while preserving physiologic function [4].

**Submucous diathermy (SMD) assisted inferior turbinoplasty:**

SMD (Submucous Diathermy) is the oldest method of treating inferior turbinate hypertrophy. Submucosal cauterization is based on coagulation of venous sinusoids within the turbinate, leading to a submucosal fibrosis and reduction of inferior turbinate volume. [5] The heat may also damage the terminal cholinergic nerve endings in reduced glandular activity.

**Fig. 2:**
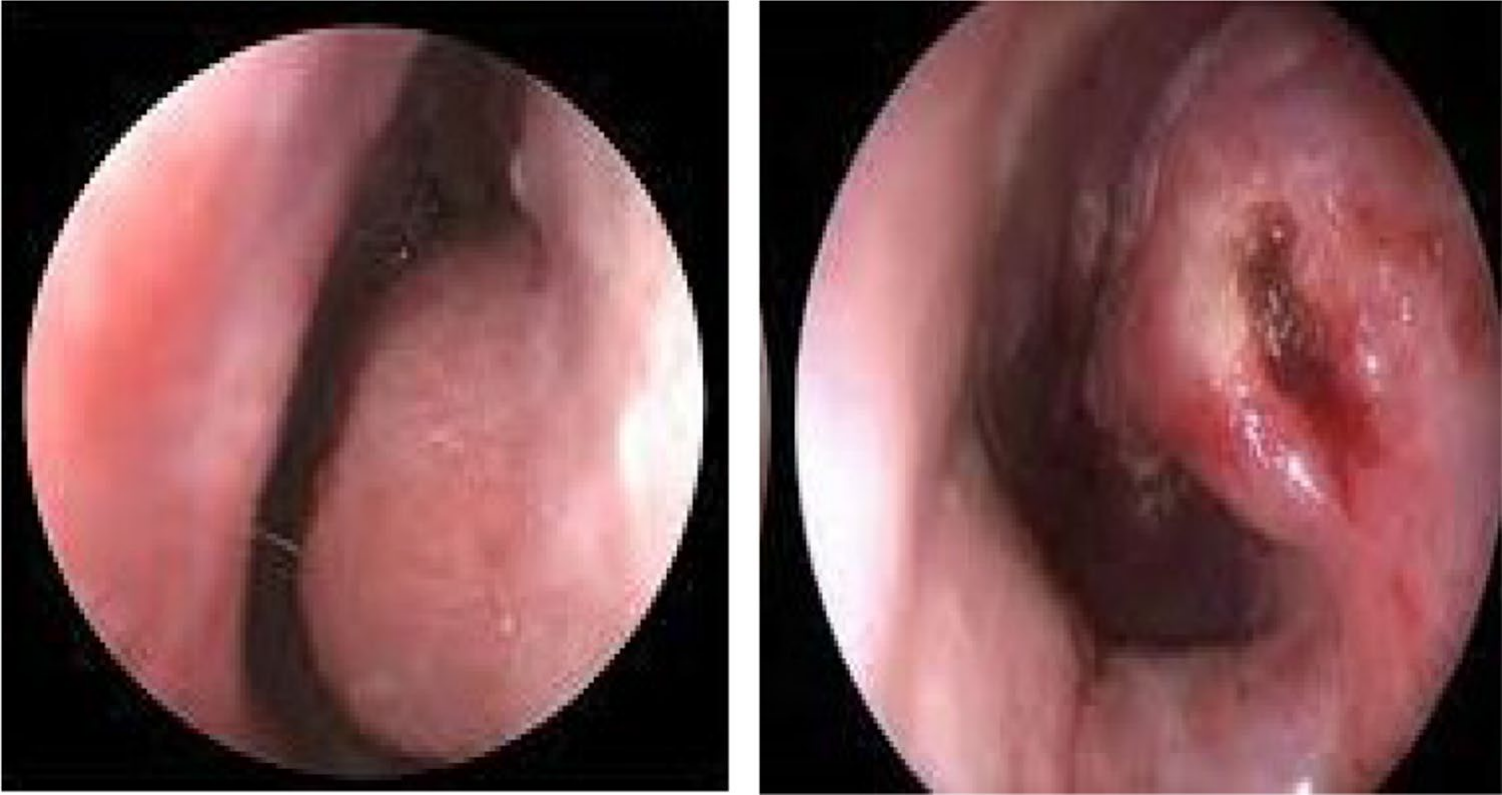
Preoperative and post-operative images of Submucous Diathermy assisted inferior turbinoplasty

*Surgical method*: This is performed by placing a spinal needle into the submucosal tissue of the turbinate longitudinally into the lower anterior part, staying parallel to but not touching the turbinate bone. An electrical current is applied for few seconds, usually till mucosal blanching is seen. Mechanism of action is hypothesized as to induce tissue destruction with vessel thrombosis & creation of scar tissue, which prevents the venous sinusoids in the turbinate mucosa from engorging [6] (Fig. 2).

**Coblation assisted inferior turbinoplasty:**

Coblation techniques utilizes a narrow electrode or wand that creates low temperature plasma of 85 degrees and effectively dissolves tissue and later induces fibrosis. A submucosal tunnel is created at the anterior inferior turbinate using coblation wand in ablation mode (power set at 7).

**Fig. 3:**
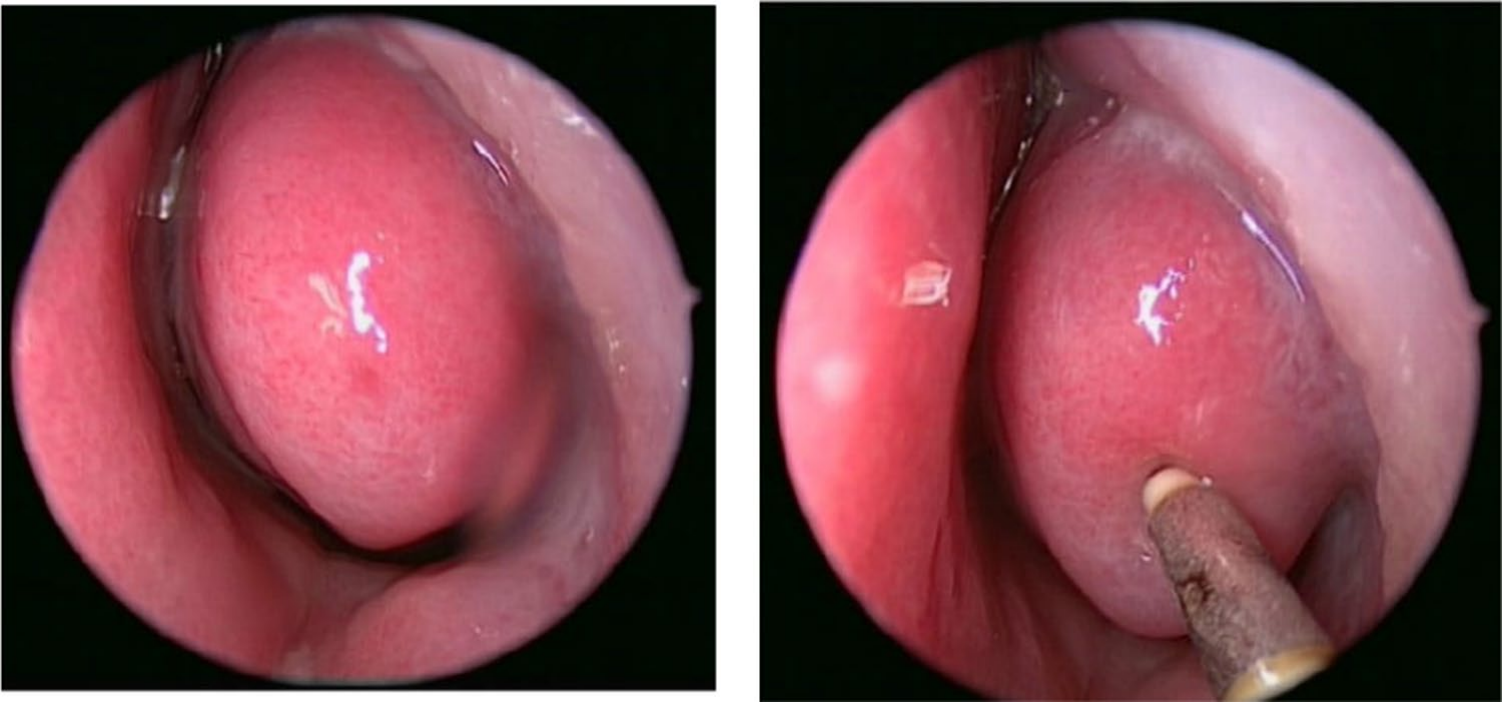
Intra-operative images of Coblation assisted Inferior turbinoplasty

*Surgical method:* Using coblation wand, the whole lateral aspect of the inferior turbinate mucosa and soft tissue is removed in anterior to posterior direction. The turbinate bone id dissected off the soft tissue using a cottle’s dissector to separate it from medial mucosa of inferior turbinate and is removed with forceps [7] (Fig. 3).

**Micro-debrider assisted inferior turbinoplasty:**

Micro-debrider is generally regarded as an essential instrument in the surgical field of rhinology. These instruments can be used on the external turbinate surface as well as intra-turbinally, often in combination with the endoscopes. This method has an advantage that they permit precise removal of soft tissue.

When **extra-turbinally** performed, only the hypertrophied portion of mucosa can be removed precisely under the direct vision of endo- scope, but damage of mucosal surface can lead to delayed healing and relatively large amount of bleeding during operation. **Intra-turbinal surgery** has an advantage of not damaging the mucosal surface. However, excessive sub- mucosal tissue may be removed and nasal packing is required for 2 to 4 days to fill the dead space within the after the procedure. [8]

**Fig. 4:**
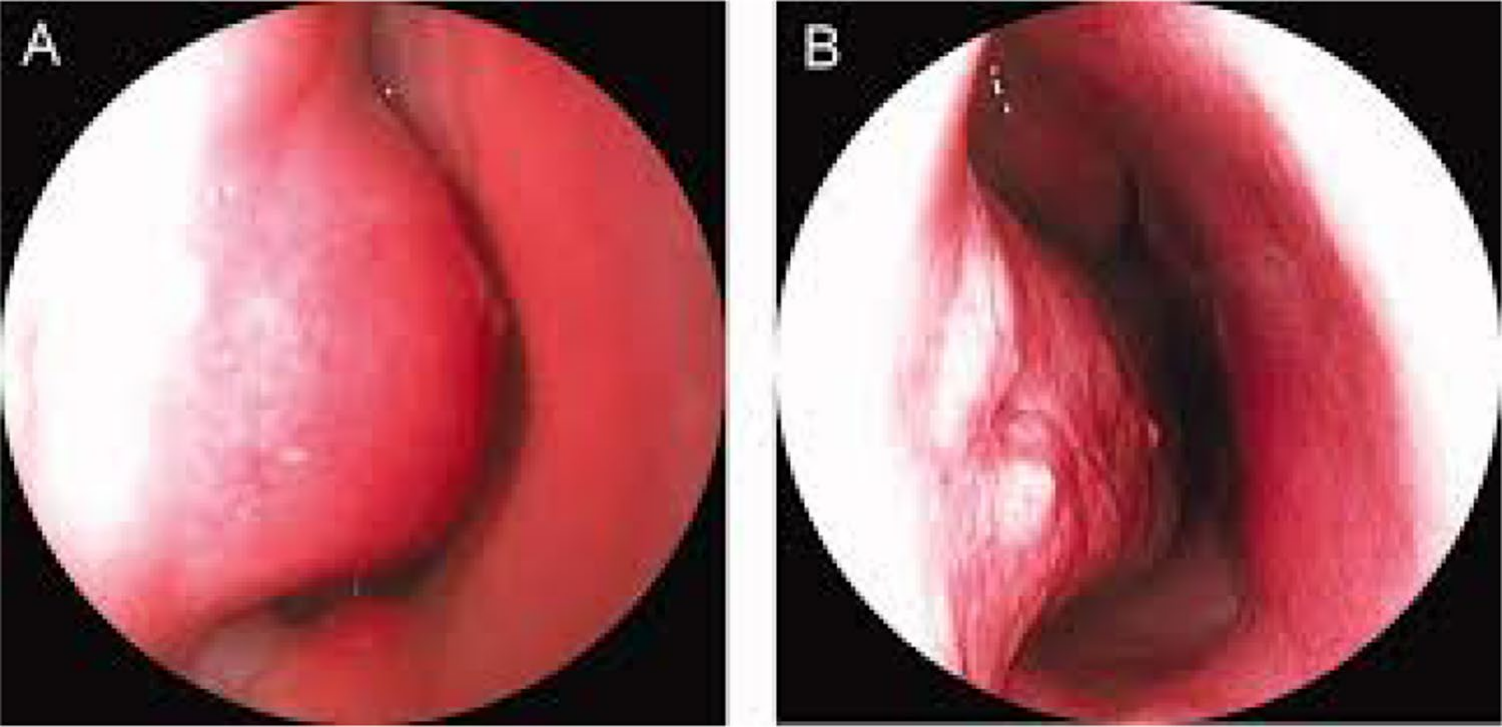
Preoperative and post-operative images of Micro-debrider assisted inferior turbinoplasty

*Surgical method*: Inferior turbinate is decongested with decongestant solution. Anterior inferior turbinate incision is placed. Micro-debrider is placed in submucosal plane and the mucosa against the concha is replaced or debrided for the reduction of the turbinate volume (Fig. 4) [2].

### Snot 22 Scoring

The sino-nasal outcomes test-22 (SNOT-22) represents the reference questionnaire to assess patients with nasal disorder. The SNOT-22 was derived from the Rhinosinusitis Outcomes Measure-31 (RSOM-31), developed by Piccirillo and co-authors. The RSOM-31 aimed to provide a holistic quantification of both health status and health-related quality of life in CRS patients in one total score. In the SNOT-20, 11 items from the RSOM31 were omitted. The resulting SNOT-22 was validated by Hopkins and colleagues [9].

### Visual Analogue Scale

The VAS (Visual analogue scale) provides a continuous scale for subjective magnitude estimation and consists of a straight line, the limits of which carry a verbal description of each extreme of the symptom to be evaluated. The line is usually 10 cm long and vertical, though different lengths and orientations have been employed and proven satisfactory. The VAS (Visual analogue scale) is often used to evaluate the analgesic properties of various treatments and accomplishes this by measuring either pain relief or pain severity. The simultaneous measurement of both has been suggested but is rarely observed. [10]

Assessment of Pain using VAS (Visual analogue scale) score: [11]

No pain 0; 2) Mild pain 1–3; 3) Moderate pain 4–7; 4) Severe Pain 8–10.

The data in this study was randomly distributed among the above mentioned three methods of inferior turbinoplasty.

As there were three groups of comparison in the present study so we have used ANOVA (Analysis of variance) test for the statistical analysis.

### Statistical Analysis with ANOVA (Analysis of Variance) Test

In the present study we have used “One way ANOVA test” for statistical analysis. One-Way ANOVA ("analysis of variance") compares the means of two or more independent groups in order to determine whether there is statistical evidence that the associated population means are significantly different. Both the One-Way ANOVA and the Independent Samples t Test can compare the means for two IT S 58 groups. However, only the One-Way ANOVA can compare the means across three or more groups. The null and alternative hypotheses of one-way ANOVA can be expressed as: H0: µ1 = µ2 = µ3 = … = µk ("all k population means are equal") H1: At least one µi different ("at least one of the k population means is not equal to the others") Where µi is the population mean of the ith group (i = 1, 2, …, k).

P value which is calculated here if < 0.05 than there is significant difference between the comparison groups. When we have not got significance in the comparison three or more groups then there is no need to compare the variance between two methods differently. If there is significance difference between the three or more groups compared, then we have to further apply test to show if there is variance in between two individual groups. [12]

## Results

See Tables 1, 2, 3, 4, 5, 6 and 7.

**Table 1:** Age distribution of patients in the present study

Age in years (n = 45)	Total no. of patients	% of patients
10–19	11	24.44
20–29	17	37.74
30–39	10	22.2
40–49	3	6.67
50–59	1	2.22
60–69	3	6.67
Total	45	100.0
Mean age	28.7 years

**Table 2:** Comparison of mean Intraoperative time (each side)

Procedure name	Mean intraoperative time in minutes (each side)		
	Present study (n = 45)	Singh study [7] (n = 33)	Jae Yong Lee study [8]
Submucous Diathermy	9.86	–	–
Coblation	8.5	10.3	4.5
Micro-debrider	11.9	8.5	4.9
P value (by ANOVA test)	< 0.0001		

As there were three groups of comparison in the present study so we have used ANOVA (Analysis of variance) test for the statistical analysis

**Table 3:** Comparison of intraoperative bleeding (each side)

Procedure name	Average Intraoperative bleeding (in ml)	
	Present study (n = 45)	S. Singh study [7] (n = 33)
Submucous Diathermy	4.06	–
Coblation	7.47	22.82
Micro-debrider	15.87	24.7
P value (by ANOVA) test)	< 0.0001	

As there were three groups of comparison in the present study so we have used ANOVA (Analysis of variance) test for the statistical analysis

**Table 4:** Comparison of average endoscopic grading

Sr. no	Procedure name	Average Endoscopic turbinate grading on follow up visits							
		Preoperatively		1 week postoperative		1 month postoperative		3 months postoperative	
		Present study	S. Singh study [7]	Present study	S. Singh study [7]	Present study	S. Singh study [7]	Present study	S. Singh study [7]
1	Submucous Diathermy	3.46	–	3.2	–	2.33	–	2.7	–
2	Coblation	3.33	3.31	3.2	2.56	2.2	1.81	1.6	1.44
3	Micro-debrider	3.6	3.24	3.2	2.36	2.2	1.48	1.46	1.3
	P value (ANOVA test)	0.699		1.0		0.634		< 0.0001	

As there were three groups of comparison in the present study so we have used ANOVA (Analysis of variance) test for the statistical analysis

**Table 5:** Distribution according to mean SNOT-22 scores

Procedure name	Mean SNOT22 score on follow up visits			
	Preoperative	1 week postoperative	1 month postoperative	3 months postoperative
Submucous Diathermy	39.33	24.46	16.33	10.4
Coblation	37.86	20.67	12.13	7.8
Micro-debrider	32.53	23.5	14.53	9.06
P value postoperatively (ANOVA test)		0.345	0.130	0.253

As there were three groups of comparison in the present study so we have used ANOVA (Analysis of variance) test for the statistical analysis

**Table 6:** Average VAS (Visual analogue score) score for postoperative pain (VAS out of 10)

Procedure name	Average VAS (Visual analogue scale) score for post-operative pain		
	After 1 week	After 1 month	After 3 months
Submucous Diathermy	7.3	5.4	3.13
Coblation	4.86	3	1.8
Micro-debrider	5.6	3.8	2
P value (ANOVA test)	< 0.001	< 0.001	< 0.001

**Table 7:** Distribution of post-operative crusting

Procedure name	No. of patients having presence of post op crusting		
	After 1 Week	After 1 Month	After 3 Months
Submucous Diathermy	9 (60%)	6 (40%)	2 (13.3%)
Coblation	5 (33.3%)	3 (20%)	1 (6.7%)
Micro-debrider	7 (46.7%)	3 (20%)	0

## Discussion

The present study involved patients between the age group 10–69 years, the mean age being 28.7 years. The incidence of Inferior turbinate hypertrophy was highest in 20–40 years of age group (i.e. 27 patients).

In the present study, the prevalence of inferior turbinate hypertrophy was marginally higher in females i.e.24 (53.4%) and the male female ratio was 0.78. This was in conformity with 55.6% females in Ahmet Erdem Kilavuz [5] study, 68% females in Mohammed A. study [13]. However, 43.9% females in Desiderio Passali study [14], 46.7% females in George Gindros study [15], 36.7% females in Jae Yong Lee study [8], 20% females in Vijay Kumar Lukka study [4], 46.7% females in M S Viahnu study [16], 38.7% females in Aamir Akbar study [17], 46% females in Smitha S. Gangaraj study [11], 42% females in Smitha Chandra B. study [6]. 3.7% females in S. Singh study [7] and 39% females in Vinay SR study [19] differs from the present study.

All the patients had complaint of Nasal blockage (i.e. 100%), 64.38% patients had complaint of Headache, 57.78% had Ear fullness and Nasal discharge, 55.55% had Disturbance of smell and 53.28% had Sneezing. No patient in the present study had complain of Nasal itching, Nasal bleeding or Snoring. Patients with enlarged inferior turbinates will invariably present with nasal obstruction [2]. In Vijay Kumar Lukka study [4], 60% patients had complaint of nasal discharge, 50% patients have complaint of sneezing, which was similar to the present study. According to Smita Chandran B. study [6], 70.4% patients had severe nasal obstruction while 29.5% patients had moderate nasal obstruction, however 54% had complaint of severe nasal discharge and 45.9% had moderate nasal discharge. In Milo Fradis study [19] 45% patients showed complaint of nasal discharge 6% patients had Headache and 39% patients had complaint of Hyposmia which was not in accordance to the present study.

26 patients (i.e. 57.8%) out of 45 patients had complaint of Nasal blockage for than 1 year followed by 14 patients (31.1%) for 6 months to 1 year and 5 patients (11.1%) for less than 6 months, with mean duration of the nasal blockage 32.7 months compared to 67.7 months in Vijay Kumar Lukka study [4].

17 patients (65.38%) out of 26 patients had complaint of Nasal discharge for more than 1 year followed by 8 patients (30.77%) for 6 months to 1 year and 1 patient (3.85%) for less than 6 months, with mean duration of nasal discharge 24.3 months.

The mean duration of nasal blockage and nasal discharge infers that most of the patients present to the hospital after a year which happens because of taking medical management from general practitioner constantly and less severity of the complaints in initial phase.

40 out of 45 patients (i.e. 88.89%) had complaint of bilateral nasal obstruction while 5 patients (i.e. 11.11%) had unilateral nasal blockage which can be attributed to the bilateral inferior turbinate hypertrophy in Allergic rhinitis and opposite inferior turbinate hypertrophy Deviated nasal septum. 20 (76.92%) out of 26 patients had complaint of bilateral nasal discharge while 6 (23.08%) patients had unilateral nasal discharge.

36 (80%) patients showed complaint of Persistent symptoms and only 9 (20%) patients showed Intermittent symptoms on elaborating history from which we can infer that chronically congested inferior turbinates not responding to medical management results in the Persistent symptoms of patients. In Vinay SR study [18] 58.3% patients had persistent nasal symptoms and 41.7% had intermittent nasal symptoms which was not in accordance with the present study.

Out of 26 patients, 24 patients (i.e. 92.31%) had Mucoid type of Nasal discharge and 2 patients (i.e.7.69%) had purulent type of nasal discharge.

On evaluating endoscopic grading of inferior turbinate preoperatively, Grade 3 was noted in 24 patients (53.33%) and Grade 4 was noted in 21 patients (46.67%). No patient showed Grade 1 and 2 preoperatively.

In the present study, the patients were diagnosed for the following four categories on the basis of anterior rhinoscopy, nasal endoscopy and computed tomographic scan of the para-nasal sinuses: 32 (71.11%) patients were diagnosed with Deviated nasal septum, followed by 16 (35.56%) patients with Allergic rhinitis, 14 (31.11%) with Chronic rhinosinusitis with or without polyposis.

Inferior turbinoplasty was done in all the 45 patients amongst which Unilateral inferior turbinoplasty was done in 30 (66.67%) patients and bilateral in 15 (33.33%) patients. Also Septoplasty in 31 (68.69%%) patients (out of which FESS was done in 9 patients), FESS was done in 14 (31.11%) patients (out of which septoplasty was done in 9 patients).

Mean intra operative time was 9.86 min in Submucous diathermy, 8.5 min in Coblation and 11.9 min in Micro-debrider assisted Inferior turbinoplasty on each side. In accordance to the present study, in S. Singh study [3], intra operative time was 10.3 min for Coblation and 8.5 min for Micro-debrider, in Jae Yong Lee study [4] the intra operative time for coblation was 4.5 min and Micro-debrider was 4.9 min.

The less intra-operative timing for Coblation and Submucous diathermy than Micro-debrider can be attributed to the powered unit used in Micro-debrider and complexity of the procedure which increase their intra-operative timing.

Average intraoperative bleeding in Submucous diathermy was 4.06 ml, 7.47 ml in coblation assisted and 15.87 ml in micro-debrider assisted inferior turbinate reduction surgery also agreeing with S. Singh study [7] findings.

There was significant difference in the average intra-operative bleeding when two methods were compared in pairs. From above data and study comparison we can say that Average Intra operative bleeding was highest in Micro-debrider assisted Inferior turbinoplasty (i.e. 15.87 ml) followed by Coblation (i.e. 7.47 ml) and Submucous diathermy (i.e. 4.06 ml) also agreeing with S. Singh study [7] findings. The higher intra-operative bleeding in Micro-debrider assisted inferior turbinoplasty can be attributed to higher amount of exposure in making mucosal flap and least thermal homeostatic effect.

On endoscopic grading pre-operatively and post-operatively we found that after 1 week post-operatively the endoscopic grading increased which subsequently decreases at 1 month and further at 3 months, which can be attributed to the post-op edema which subsides after 1 week.

On comparing the three surgical methods we found that endoscopic grading improved more in patients who had undergone Micro-debrider assisted inferior turbinoplasty followed by Coblation and Submucous diathermy which shows the symptom improvement and level of efficacy of the surgical methods.

We compared Average endoscopic grading of the inferior turbinate according to the Camacho [3] classification preoperatively and postoperatively we found that average grading decreased on the follow up visits which was in accordance to S. Singh study [7].

On applying ‘one-way ANOVA test, there was no significant difference between the average turbinate grading on preoperatively and follow up after 1 week and 1 month. But there was significance difference in the average endoscopic grading at 3 months postoperatively.

This suggest that after 3 month the endoscopic grading decreases significantly in Micro-debrider and Coblation assisted inferior turbinoplasty compared to Submucous Diathermy which can be attributed to the conventional and less powered approach used for Submucous diathermy.

In the present study, we have taken pre-operative and post- operative SNOT 22 (Sino nasal outcome test) score from each patient which suggested that SNOT 22 Score decreases on follow up visits due to improvement in nasal airflow and reduction and healing of the Inferior turbinate. According to the post-operative SNOT 22 scoring, best improvement in symptoms is seen in Inferior turbinate reduction with Coblation followed by Micro-debrider and Sub mucous diathermy at the end of 3 months.

On applying ‘one-way ANOVA’ test, there is no significant difference between the SNOT22 scores measured postoperatively for the three surgical methods which signifies that there is no significant difference in the postoperative improvement in the symptoms between the inferior turbinoplasty done with Submucous diathermy, Coblation and Micro-debrider at the end of 1 week, 1 month and 3 months.

In the present study, Average VAS score at post-operative visit after 1 week in Submucous diathermy was 7.3 which decreased to 5.4 and 3.13 on further post op visits at 1 month and 3 months respectively. In Coblation average VAS score was 4.8 after 1 week which decreases to 3 and 1.8 after 1 month and 3 months respectively. In Micro-debrider, average VAS score was 5.6 after 1 week which decreases to 3.8 and 2 after 1 month and 3 months respectively. So we can say that Average VAS score for post-operative pain kept decreasing on follow up visits. Least score was found with Coblation assisted inferior turbinoplasty i.e. 1.8 at the end of 3 months.

There was significant difference in average postoperative VAS score when Submucous diathermy was compared with Coblation and Submucous diathermy with Micro-debrider individually, but there was no significant difference in average postoperative VAS score when Coblation was compared with Micro-debrider at the end of 1 week, 1 month and 3 months. The difference and improvement of the VAS score in Coblation at follow up visits can be attributed to the mechanism of action i.e. by least thermal effect and least peripheral tissue damage.

In this present study, average post-operative VAS after 1 week with coblation was 4.86 and 5.6 with micro-debrider assisted turbinoplasty. In S. Singh study [7] average post-operative VAS after 1 week with coblation was 2.19 and 2.65 with micro-debrider assisted turbinoplasty which was in accordance to the present study. In Mohammed A. Gomma study [13], 44% of patients who has Submucous Diathermy had moderate post op pain (VAS score of 4–7) and 56% had mild post-operative pain (VAS score 1–3) after 2 weeks. 4 patients (i.e. 13.3%) had complaint of nasal pain on day 1 postoperatively and no patient after 1 week in M S Vishnu study [16].

In the present study, we infer that Post-operative crusting decreases on follow visits with highest incidence seen in Submucous Diathermy followed by Micro-debride and least seen in Coblation assisted inferior turbinoplasty. After 1 week, 60% patients showed post-operative crusting in the present study, 33.3% patients in Vinay S R study [18], 16.7% patients in Vishnu study[16] and 37.3% in Aamir Akbar study[17] which subsequently decreased on follow-up at 1 month and 3 months. In Milo Fradis study [19], 6% patients showed nasal crusting. In Desiderio Passali study [14] 39 patients (i.e. (62.9%) showed post-operative crusting.

*Post-operative Synechiae formation* was seen only in 1 patient with Inferior Turbinoplasty by Coblation method in this study. In M S Vishnu study [16] none of the patients had synechiae formation, in Desiderio Passali study [14], 21 patients (i.e. 33.8%) patients showed post-operative synechiae formation.

## Conclusion

Out of 45 patients, highest number of patients belonged to 20–40 years of age group (i.e.27 patients) with the mean age 28.7 years. The prevalence of the inferior turbinate hypertrophy was seen marginally higher in female (53.38%) than males and the male female ratio being 0.78:1.

Most of the patients had Bilateral Nasal blockage and Nasal discharge (mucoid type) for more than a year duration.

The above mentioned three surgical were compared on the basis of preoperative, intraoperative and postoperative parameters like SNOT 22 scores, intraoperative timing and bleeding, Endoscopic grading and VAS score for post-operative pain. The comparison among the three methods showed the variation in all parameters according to the mechanism of action and the powered instrument used for surgery.

From the present study we conclude:

- On comparing all the above methods, we found that Coblation and Micro- debrider were more or less equally effective and better than Sub-mucous diathermy for inferior turbinoplasty. There was no significant difference in the various parameters measured for Coblation and Micro-debrider.
- Coblation assisted turbinoplasty is better in terms of intra-operative bleeding and timing and post-operative pain when compared to Micro-debrider. While micro-debrider is better in terms of endoscopic grade and symptomatic turbinate improvement and post-operative crusting when compared to Coblation.
- Although there was significant difference when Coblation/ Micro-debrider was compared to Sub-mucous diathermy. Submucous diathermy has least benefits, still most commonly used method because of its simplicity, conventionality and least cost factor while other two methods need capital investment and higher learning curve of the surgeon.
- The selection of the type of method still remains in the hand of the operating surgeon which depends on the associated pathology, patient comfort and affordability of the hospital and patient.

### Limitations of the Present Study

- The presence of associated other nasal pathologies influenced the scores and results.
- The preference of the type of method is based on the surgeon’s choice and leaning curve in most cases.
- Though Coblation and Micro-debrider are more advanced still less preferred than Sub-mucosal diathermy due to its simplicity and feasibility.
